# An unusual indication for splenectomy in hairy cell leukaemia: a report of three cases with persistent splenomegaly after chemoimmunotherapy

**DOI:** 10.1111/bjh.13767

**Published:** 2015-09-25

**Authors:** Nadav Sarid, Humayun N Ahmad, Andrew Wotherspoon, Claire E Dearden, Monica Else, Daniel Catovsky

**Affiliations:** ^1^ Tel Aviv Sourasky Medical CentreTel AvivIsrael; ^2^ Queen's HospitalBurton upon TrentUK; ^3^ The Royal Marsden NHS Foundation TrustLondonUK; ^4^ Division of Molecular PathologyThe Institute of Cancer ResearchLondonUK

**Keywords:** hairy cell leukaemia, splenomegaly, splenectomy, chemoimmunotherapy, spleen histology

## Abstract

We describe three cases of relapsed hairy cell leukaemia (HCL) treated with pentostatin plus rituximab. All three achieved bone marrow complete remission but had persistent splenomegaly and hypersplenism. Because of the clinical uncertainty of its significance, they were all splenectomized. The spleen histology showed no evidence of HCL, but a five‐fold thickening of the splenic capsule and areas of fibrosis in the red pulp. This process may have contributed to the lack of elasticity and caused the persistent splenomegaly. We discuss the clinical implications for future patient management. The three patients remain in remission at 1 + , 5 +  and 9 +  years.

## Introduction

Purine analogues have revolutionized the treatment of hairy cell leukaemia (HCL) with overall responses in more than 85% of patients and a median progression‐free survival (PFS) of up to 15 years (Else *et al*, 2009; Grever, 2010).

Despite the success of pentostatin and cladribine, PFS curves have revealed no plateau in long‐term follow‐up studies (Else *et al*, 2009). In addition, there is a small subset of patients with primary refractory disease.

Second and third line treatment with single‐agent purine analogues produce lower complete remission (CR) rates and shorter PFS (Chadha *et al*, 2005). In that context, the combination of pentostatin or cladribine with rituximab was tested and found to be effective (Else *et al*, 2011), and is now recommended in the UK for recurrent or refractory HCL (Jones *et al*, 2012).

Here we describe three patients with relapsed HCL treated with pentostatin and rituximab, with a seeming discrepancy between a documented CR in the bone marrow (BM) and a non‐resolved splenomegaly. The patients also had continuing thrombocytopenia. The possibility of residual HCL in the spleen was raised and all three patients underwent splenectomy.

## Patients

### Case 1

A man aged 49 years was diagnosed with HCL in June 2003. He presented with a large spleen, thrombocytopenia and circulating hairy cells (15 × 10^9^/l). He was treated with cladribine for 5 days with a good clinical response. After treatment, a BM biopsy showed 4% residual HCL cells by immunostaining. He was then referred to the Royal Marsden Hospital, London. He was clinically well and had no physical signs, with normal blood counts. In January 2006 there was evidence of progression: the platelet count had dropped to 52 × 10^9^/l and an ultrasound scan revealed that the spleen was enlarged (21·3 cm). A BM aspirate showed 10% HCL cells and the trephine biopsy was consistent with relapsed HCL. He was then treated with a combination of pentostatin (4 mg/m^2^ for 6 doses) and rituximab (375 mg/m^2^ for 4 doses) every 2 weeks, given concurrently.

The BM biopsy at the end of treatment showed CR, with no evidence of residual HCL, but the platelet count was still low (80 × 10^9^/l) and the spleen remained enlarged (17 cm). A laparoscopic splenectomy was performed 6 weeks after completion of the chemoimmunotherapy. The blood counts normalized rapidly and nine years later he remains asymptomatic with normal counts (Table 1).

**Table 1:** Blood counts before and after treatment

Results	Before pentostain plus rituximab	After pentostatin plus rituximab (prior to splenectomy)	After splenectomy	Current
Haemoglobin (g/l)				
Case 1	155	150	150	157
Case 2	123	145	139	141
Case 3	132	129	134	143
White blood cell count (× 10^9^/l)				
Case 1	2·0	3·8	5·6	9·9
Case 2	1·7	2·5	5·7	4·2
Case 3	3·7	5·0	11·6	10·7
Platelet count (× 10^9^/l)				
Case 1	52	80	380	403
Case 2	36	67	281	342
Case 3	45	107	1257	656

### Case 2

A man aged 48 years was diagnosed with HCL in 1999 and received treatment with cladribine for 5 days. He remained clinically well for 5 years. In 2006 he needed to be retreated with cladribine after relapse. His response was documented as a partial response on BM testing.

In 2009, aged 59 years, he developed fullness on the left side of his abdomen. His platelet count was 36 x 10^9^/l and a computerized tomography (CT) scan showed a grossly enlarged spleen (29 cm in the craniocaudal axis). A BM biopsy showed HCL with 60% infiltration. He was treated at Queens Hospital, Burton upon Trent, with a combination of pentostatin (4 mg/m^2^ for 8 doses) and rituximab (375 mg/m^2^ for 6 doses) every two weeks concurrently from April to July 2010. At the end of treatment the BM biopsy was consistent with CR, with no evidence of HCL, but the platelet count remained low (67 × 10^9^/l) and a CT scan in October 2010 showed the spleen was still enlarged (21 cm). As there was uncertainty about the response, a laparoscopic splenectomy was performed 5 months after completion of treatment.

Five years later he remains asymptomatic with normal blood counts (Table 1).

### Case 3

A woman aged 43 years was diagnosed with HCL in April 2013 and was treated with intravenous cladribine for 5 days in the Tel‐Aviv Sourasky Medical Centre. After treatment a BM biopsy showed 5% residual HCL cells and in the subsequent months there was symptomatic enlargement of the spleen (21 cm) and a decrease in platelet count to 45 × 10^9^/l. A repeat BM biopsy demonstrated 40% HCL cells. In March 2014, treatment with a combination of pentostatin (4 mg/m^2^ for 9 doses) and rituximab (375 mg/m^2^ for 8 doses) was given concurrently every 2 weeks. After 7 cycles of treatment a repeated BM biopsy showed no evidence of HCL, but the platelet count was low (107 × 10^9^/l). Eight weeks after completion of the chemoimmunotherapy an abdominal magnetic resonance imaging scan demonstrated that the spleen was still bulky, measuring 15 cm in both the axial and coronal planes. A laparoscopic splenectomy was performed. The spleen weight was 800 g.

A year later the patient is still in remission with normal blood counts (Table 1).

## Spleen histology

**Figure 1:**
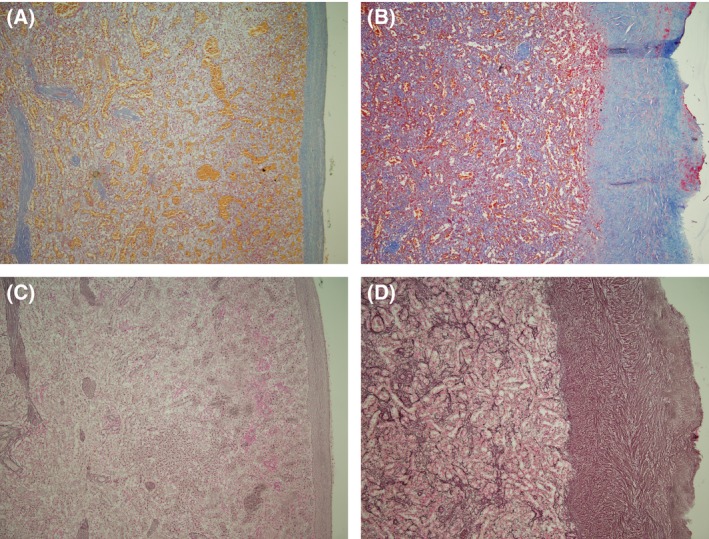
Spleen histology. A & B Martius scarlet blue stain for collagen. (A) Normal spleen (control); (B) Spleen of Case 1. C & D: Reticulin stain. (C) Normal spleen (control); (D) Spleen of Case 1. Panels B & D illustrate clearly the fivefold thickening of the spleen capsule. All panels taken at low power magnification.

Local pathologists initially reported that the spleens of the three patients showed no evidence of residual HCL, which was subsequently confirmed by one of the authors. In addition, the following observations were made. The sections were negative for CD20 and TRAP by immunohistochemistry. There was extensive loss of the white pulp with residual periarteriolar cuffs of T‐cells. The main finding was a fivefold thickening of the splenic capsule compared with a normal spleen, shown by stains for collagen (Fig 1A and B). The measurements of the capsule were as follows: 540 μm (Case 1), 497 μm (Case 2) and 637 μm (Case 3) compared with 111·56 + /‐ 21·45 μm in a published control series of 30 spleens (Alim *et al*, 2012). There were also areas of patchy fibrosis in the red pulp and around the sinusoids, shown by reticulin staining. A comparison was made with normal spleen tissue, which did not show those changes (Fig 1C and D).

## Discussion

HCL patients achieving a CR after treatment have significantly longer PFS than those achieving only a partial response (Else *et al*, 2009). Therefore, the guidelines for the management of HCL (Jones *et al*, 2012) have emphasized the need to maximize responses in order to prolong remissions, and the importance of accurate assessment of response after treatment. CR is defined as the absence of hairy cells from the peripheral blood and BM biopsy, along with resolution of organomegaly and cytopenias (Jones *et al*, 2012).

The intriguing clinical scenario we describe of persistent splenomegaly with hypersplenism manifesting as thrombocytopenia can hamper accurate assessment of response. As pentostatin is usually administered until maximum response and then two more doses beyond as consolidation (Else *et al*, 2011), this phenomenon may expose the patient to an unnecessary amount of chemotherapy if they are not considered to be in CR.

Splenectomy was decided upon because of residual splenomegaly, hypersplenism and clinical uncertainty concerning the response achieved by the pentostatin and rituximab combination. The persistence of splenomegaly after a successful BM response could also be caused by infection even in the absence of fever. All three splenic specimens showed no HCL or infection but increased collagen and reticulin around vessels in the red pulp and in the capsule. The capsule showed a fivefold thickening compared with normal spleens (Alim *et al*, 2012).

All three cases, two in first relapse (Cases 1 and 3) and one in second relapse (Case 2), had significantly large spleens prior to treatment with pentostatin and rituximab. HCL is invariably associated with BM as well as splenic fibrosis, which is caused by the formation of a fine network of reticulin fibres (Shehata *et al*, 2004). Collagen fibres can also be observed in the advanced stages of the disease. High expression levels of *TGFB1* RNA and TGF‐β1 (TGFB1) protein have been demonstrated in blood, spleen and BM cells in HCL and hairy cells have been shown to represent the major source of TGF‐β1. In addition, TGF‐β1 is directly involved in enhancing the synthesis of reticulin fibres by BM fibroblasts in HCL (Shehata *et al*, 2004).

As there was no residual leukaemia in the spleen, it would appear that the persistent splenomegaly was caused by loss of elasticity secondary to capsular and red pulp fibrosis. It is not clear whether the splenomegaly would have regressed spontaneously over time, however we thought that a strategy leaving a potentially infiltrated spleen in place was risky, especially considering that the BM had no evidence of disease and therefore CR might be within reach.

Two of our patients (Cases 1 and 2) remain in a long lasting remission, 9 and 5 years after splenectomy. Case 3 is also still in remission albeit with a shorter follow‐up period. It is not possible to know what would have happened if the spleens were left in‐situ as responses with pentostatin and rituximab are generally very good (Else *et al*, 2011). However, it is well known that splenectomy is an effective treatment in a minority of HCL patients, especially those who have predominantly splenic involvement, resulting in prolonged remissions (Bouroncle *et al*, 1958; Ng *et al*, 1987).

In conclusion, although splenectomy is no longer performed as a standard of care in HCL, these three cases demonstrate that, even in the era of purine analogues and immunotherapy, splenectomy may be useful as a means to determine outcome. This report draws attention to the phenomenon of splenomegaly without disease involvement and helps spare patients in CR from further potentially harmful chemotherapy.

## Competing interests

The authors have no competing interests.
